# Flux balance analysis accounting for metabolite dilution

**DOI:** 10.1186/gb-2010-11-4-r43

**Published:** 2010-04-16

**Authors:** Tomer Benyamini, Ori Folger, Eytan Ruppin, Tomer Shlomi

**Affiliations:** 1 The Blavatnik School of Computer Science, Tel Aviv University, Tel Aviv 69978, Israel; 2 The Sackler School of Medicine, Tel Aviv University, Tel Aviv 69978, Israel; 3 Computer Science Department, Technion - Israel Institute of Technology, Haifa 32000, Israel

## Abstract

Flux balance analysis is a common method for predicting steady-state flux distributions within metabolic networks, accounting for the growth demand for the synthesis of a predefined set of essential biomass precursors. Ignoring the growth demand for the synthesis of intermediate metabolites required for balancing their dilution leads flux balance analysis to false predictions in some cases. Here, we present metabolite dilution flux balance analysis, which addresses this problem, resulting in improved metabolic phenotype predictions.

## Abstract 2

A flux balance analysis method for gene essentiality prediction, which takes into account variation in biomass composition under different growth conditions.

## Background

A practical approach to gaining biological understanding of complex metabolic networks requires the development of mathematical modeling, simulation, and analysis techniques. Traditional modeling techniques are based on mathematical approaches that require detailed and accurate information regarding reaction kinetics as well as enzyme and metabolite concentrations [1,2]. The lack of sufficient data limits the current applicability of such methods to small-scale systems. This hurdle is surpassed through the use of constraint-based modeling (CBM), which serves to analyze the functionality of genome-scale metabolic networks by relying solely on simple physical-chemical constraints [3,4]. Genome-scale CBM models have already been constructed for more than 50 organisms [5], including common model microorganisms [6,7], industrially relevant microbes [8-11], various pathogens [12-15], and recently for human cellular metabolism [16]. Flux balance analysis (FBA) is a key computational approach within the CBM modeling framework [17-19] and is frequently used to successfully predict various phenotypes of microorganisms, such as their growth rates, uptake rates, by-product secretion, and knockout lethality (see [3,5,20] for reviews).

Traditional kinetic models of cellular metabolism are formulated as a set of differential equations that compute the time derivative of metabolite concentrations (denoted by ) as dependent on reaction rates (denoted by ; which, in turn, depend on metabolic concentration and kinetic constants, denoted by ) and metabolite dilution due to cellular growth (with *μ *denoting the growth rate) [21]:(1)

where *S *is an *m *× *n *stoichiometric matrix, *m *is the number of metabolites, *n *is the number of reactions, and *S*_*ij *_represents the stoichiometric coefficient of metabolite *i *in reaction *j*. A precise solution to Equation 1 requires determination of the kinetic parameters , which are generally unavailable, resulting in the development of the alternative CBM approach. In CBM, an entire space of possible solutions for the flux distribution  is postulated, considering that the metabolic system is constrained by physicochemical, environmental and regulatory constraints. In FBA, this solution space is constrained by the assumption of a quasi steady-state, under which stoichiometric mass-balance constraints enforce constant concentrations of intermediate metabolites over time:(2)

The uptake and secretion of a pre-defined set of metabolites from and to the environment is facilitated via the definition of exchange reactions in the stoichiometric matrix *S *[3]. A pseudo growth reaction is defined to simulate the utilization of metabolites during growth, consuming the most abundant biomass constituents based on experimentally determined concentrations (that is, the *j*-th component in  denotes the steady-state concentration of metabolite *j*). The objective of FBA is to find a steady-state flux distribution, , satisfying Equation 2 alongside additional enzymatic directionality and capacity constraints [3], together permitting a maximal growth rate *μ*. Accounting only for linear constraints, the resulting space of feasible flux distribution described by FBA is convex (forming a high-dimensional polytope), in which optimal biomass producing solutions can be efficiently searched for via linear programming (LP).

The employment of a pseudo growth reaction in FBA to represent the utilization of metabolites as part of growth poses two fundamental problems. First, the metabolite composition of cellular biomass significantly varies across different growth media, genetic backgrounds and growth rates [22-24]. Indeed, previous work by Pramanik and Keasling [22,23] has shown that using the correct experimentally measured biomass composition of *Escherichia coli *under different growth media and growth rates significantly improves FBA flux predictions. However, as FBA is commonly applied to probe metabolic behavior under diverse genetic and environmental conditions for which no metabolite concentration data are available, it has become common practice to employ a constant biomass composition across all conditions [25]. Second, the growth reaction in various CBM models commonly accounts for no more than a few dozen metabolites, for which measured concentrations are available under a specific condition [23]. Ignoring the growth-associated dilution of the remaining metabolites (those not included in the biomass composition in use; required by Equation 1) may result in the prediction of biologically implausible flux distributions, leading to false predictions of gene essentiality and growth rates, as shown in the Results. This problem has been recently addressed by Kruse and Ebenhöh [26], who suggested a method that is based on network expansion to compute the set of producible metabolites under a given growth medium. This method, however, does not enable the prediction of feasible flux distributions that account for the growth-associated dilution of all intermediate metabolites. Another approach, recently suggested by Martelli *et al. *[27], predicts metabolic fluxes based on Von Neumann's model, which maximizes the growth rate in a metabolic network without assuming mass-balance nor utilizing prior knowledge of a biomass composition. However, similarly to FBA, flux distributions predicted by this method do not fully account for the growth-associated dilution of all intermediate metabolites.

In this paper we describe a variant of FBA, metabolite dilution flux balance analysis (MD-FBA), which aims to predict metabolic flux distributions by accounting for the dilution of all intermediate metabolites that are synthesized under a given condition. As shown below, accounting for growth dilution of intermediate metabolites is especially important for metabolites that participate in catalytic cycles, many of them being metabolic co-factors. Since CBM assumes a steady-state flux distribution and does not predict the actual concentration of the intermediate metabolites, we consider a uniform minimal dilution rate for all intermediate metabolites produced via a non-zero flux through some reaction (assuming a uniform concentration for all intermediate metabolites, following [28]).

**Figure 1:**
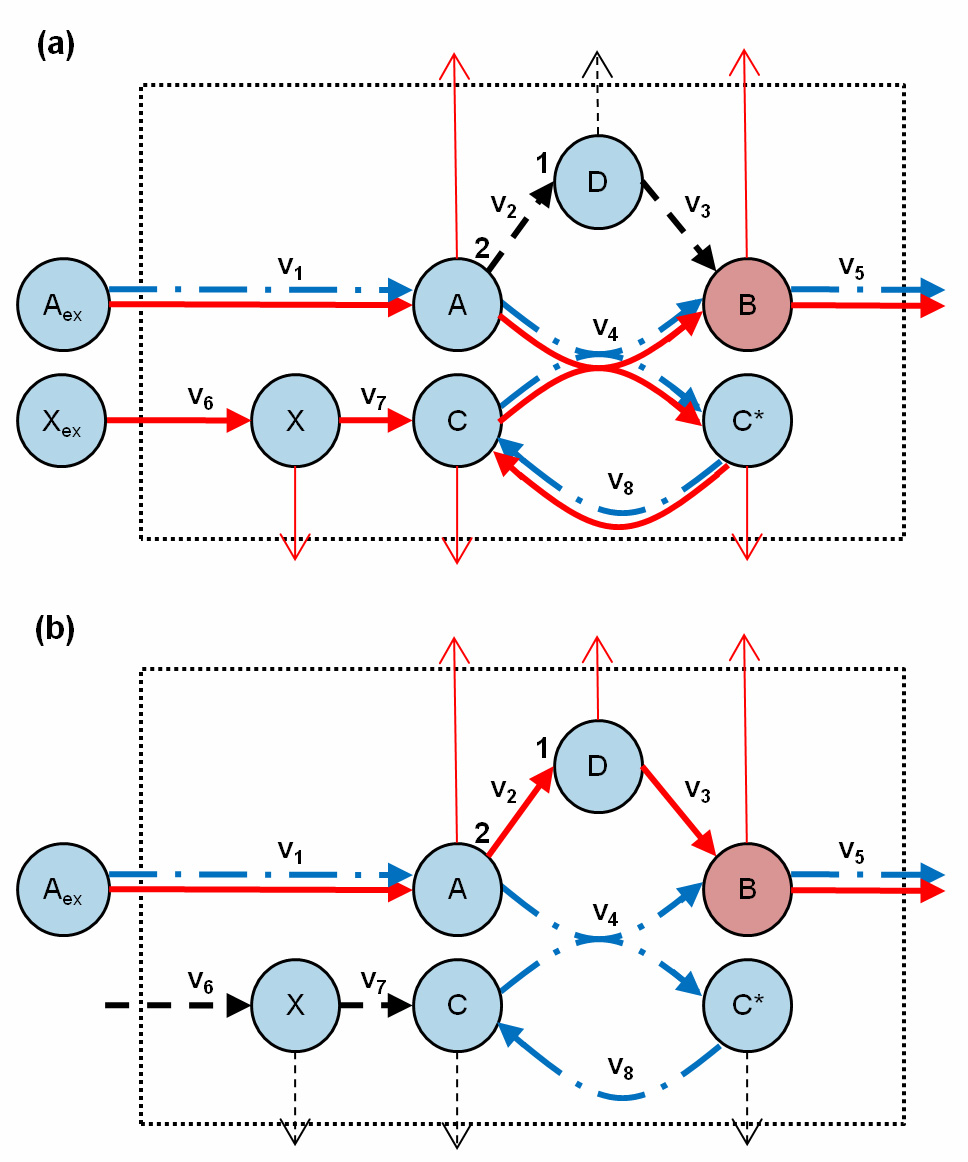
**An example network featuring the difference in predicted flux distributions between FBA and MD-FBA**. Thick arrows represent metabolic reactions and circular nodes represent metabolites. Narrow arrows represent the growth-associated dilution of their attached metabolites. Note that the stoichiometric coefficients for reaction *v*_2 _are two molecules of A per one molecule of D. *v*_1 _and *v*_6 _represent the uptake for metabolites *A *and *X*, respectively. *B *is the sole metabolite within the biomass, and hence the flux through *v*_5 _represents the growth rate. Blue (dash-dot) and red (solid) arrows represent reactions predicted to be active by MD-FBA and FBA, respectively, while black (dashed) arrows represent all other reactions. The figure illustrates growth on two media: **(a) **growth on a medium in which both *A *and *X *are present; **(b) **growth on a medium including only metabolite *A*. FBA predicts the same growth rate, which is equal to *v*_1 _under both media, while MD-FBA predicts a growth rate equal to *v*_1 _when both *A *and *X *are present in the medium and a growth rate equal to 0.5v1 when only A is included in the medium. The latter is due to the fact that when *X *is absent from the growth medium, MD-FBA cannot activate reactions *v*_4 _and *v*_8_, since the dilution of metabolites *C *and C* cannot be satisfied under this medium. The different flux distributions predicted by the two methods lead to different predictions of enzyme essentiality, as detailed in the main text.

Figure 1 demonstrates an example network for which FBA and MD-FBA predict different flux distributions, leading to different growth rate and gene essentiality predictions. The biomass in this network is metabolite *B*, while the input metabolites available in the growth medium are *A *and *X *in Figure 1a, and only A in Figure 1b. The synthesis of the biomass precursor *B *is facilitated via two alternative pathways: through an efficient pathway via *v*_4_, producing one molecule of *B *per molecule of *A*; or through an inefficient pathway via reactions *v*_2 _and *v*_3_, producing one molecule of *B *per two molecules of *A*. Reaction *v*_4 _requires the presence of a co-factor metabolite *C*, which is recycled via reaction *v*_8 _and synthesized via reactions *v*_6 _and *v*_7_. Thus, in MD-FBA, the activation of the efficient pathway for synthesizing *B *via *v*_4 _enforces *de novo *synthesis of co-factor *C *via *v*_6 _and *v*_7 _to balance the dilution of this co-factor (Figure 1a, red solid arrows). By contrast, since FBA does not account for metabolite dilution, it would predict a biologically implausible flux distribution in which the steady-state concentration of the co-factor *C *is maintained via reaction *v*_8_, without predicting the growth-associated demand for *de novo *synthesis of this co-factor (Figure 1a, b, blue dot-dash arrows). The different flux distributions predicted by FBA and by MD-FBA under the two growth media yield different growth rate and enzyme essentiality predictions. FBA predicts the activation of the efficient biosynthetic pathway for synthesizing metabolite *B *under both growth media, resulting in the same growth rate prediction under the two media. MD-FBA, on the other hand, predicts the activation of the efficient biosynthetic pathway when metabolite *X *is present in the growth medium (with a growth rate prediction similar to that of FBA; Figure 1a) and the activation of the inefficient pathway when metabolite *X *is absent (resulting in a lower growth rate; Figure 1b). When metabolite *X *is present in the growth medium, MD-FBA, unlike FBA, predicts that the biosynthetic pathway for the production of co-factor *C *is activated, with the reactions *v*_6 _and *v*_7 _being essential for achieving maximal growth rate (Figure 1a). When *X *is absent from the growth medium, FBA predicts the activity of the efficient pathway through *v*_4 _and *v*_8_, with the corresponding enzymes essential for obtaining a maximal growth rate. MD-FBA, however, predicts the inactivation of this efficient pathway and hence the inessentiality of *v*_4 _and *v*_8_, while predicting the enzymes in the less efficient pathway *v*_2 _and *v*_3 _to be essential for growth (Figure 1b).

Next, we describe the implementation of MD-FBA as a mixed-integer linear programming (MILP) optimization problem and demonstrate its applicability in predicting metabolic phenotypes, outperforming the commonly used FBA method.

## Results

### MD-FBA - accounting for growth-associated dilution of all intermediate metabolites

Our method, MD-FBA, aims to predict a feasible flux distribution through a metabolic network under a given environmental and genetic condition, by maximizing the production rate of the biomass (that is, the flux through the biomass reaction) while satisfying a stoichiometric mass-balance constraint, accounting for the growth-associated dilution of all produced intermediate metabolites, and satisfying enzymatic directionality and capacity constraints embedded in the model (similarly to FBA). MD-FBA is formulated as a MILP problem as defined in the Materials and methods.

### Applying MD-FBA to predict metabolic phenotypes in *Escherichia coli*

As a benchmark for the prediction performance of MD-FBA, we applied it to the genome-scale metabolic network model of *E. coli *[6] to predict growth rates and gene essentiality under a diverse set of growth media and gene knockouts. The model of Feist *et al. *[6] accounts for 1,260 metabolic genes, 2,382 reactions and 1,668 metabolites.

**Figure 2:**
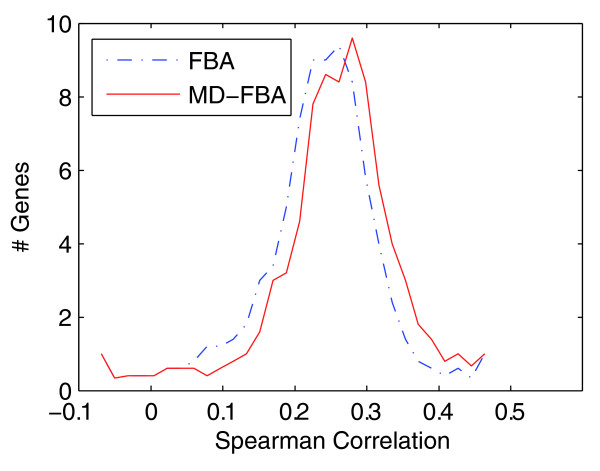
**Histograms of Spearman correlation values between measured and predicted growth rates**. The histograms show the accuracy of FBA (blue, dash-dot line) and MD-FBA growth rate predictions (red, solid line) for 81 gene deletion strains across 96 growth media. The median Spearman correlation for MD-FBA is significantly higher than that of FBA (Wilcoxon test *P*-value = 0.0145).

As an initial validation, we applied both MD-FBA and FBA to predict *E. coli*'s growth rate for 91 gene knockout strains under 125 different media, yielding a total of 11,375 growth conditions for which measured optical density (OD) data are available via a phenotypic array in the ASAP database [29]. Each medium included a fixed set of metabolites (oxygen, phosphate, water, sulfate, carbon dioxide, hydrogen and metal ions) and alternating carbon and nitrogen sources (the full list of growth conditions (media and gene knockouts) as well as the experimental OD values are available in Additional files 1 and 2, respectively). Different gene knockouts were modeled by forcing a zero flux through the corresponding enzyme-catalyzed reactions [28]; different growth media were modeled by changing the bounds on metabolite uptake from the environment based on specification of the available metabolic nutrients in each medium [28]. Both FBA and MD-FBA predicted no growth for the wild-type strain under 13 growth media and hence these media were removed from further analysis. In an additional 16 growth media the correlation between the growth rates predicted by FBA and MD-FBA across all knockout strains was significantly high (Spearman *r *> 0.7) and hence these media were also removed from further comparison of the two methods (the results presented below are insensitive to specific choice of a Spearman correlation threshold). For each deletion strain, a Spearman correlation was calculated between the predicted growth rates and the measured OD values across the remaining 96 different growth media. For 10 of the 91 gene deletion strains, both FBA and MD-FBA falsely predicted zero growth across all media and these strains were removed from further analysis. The median Spearman correlation obtained by MD-FBA was found to be slightly higher than that obtained via FBA (Wilcoxon test *P*-value = 0.0145; Figure 2). Several limitations of the MD-FBA method currently restrict its ability to markedly improve the growth rate predictions, as discussed below. Still, in some interesting specific cases MD-FBA outperforms FBA; for example, we examined two minimal growth media, N-acetyl-D-mannosamine and N-acetyl-D-glucosamine, under which the latter yields a higher measured growth rate across all knockout strains, while FBA predicted identical growth rates for all 81 knockout strains under both media. MD-FBA, on the other hand, predicted different growth rates for 67 of the knockout strains under the two growth media, correctly predicting a higher growth rate in N-acetyl-D-glucosamine in 87% of these cases.

**Figure 3:**
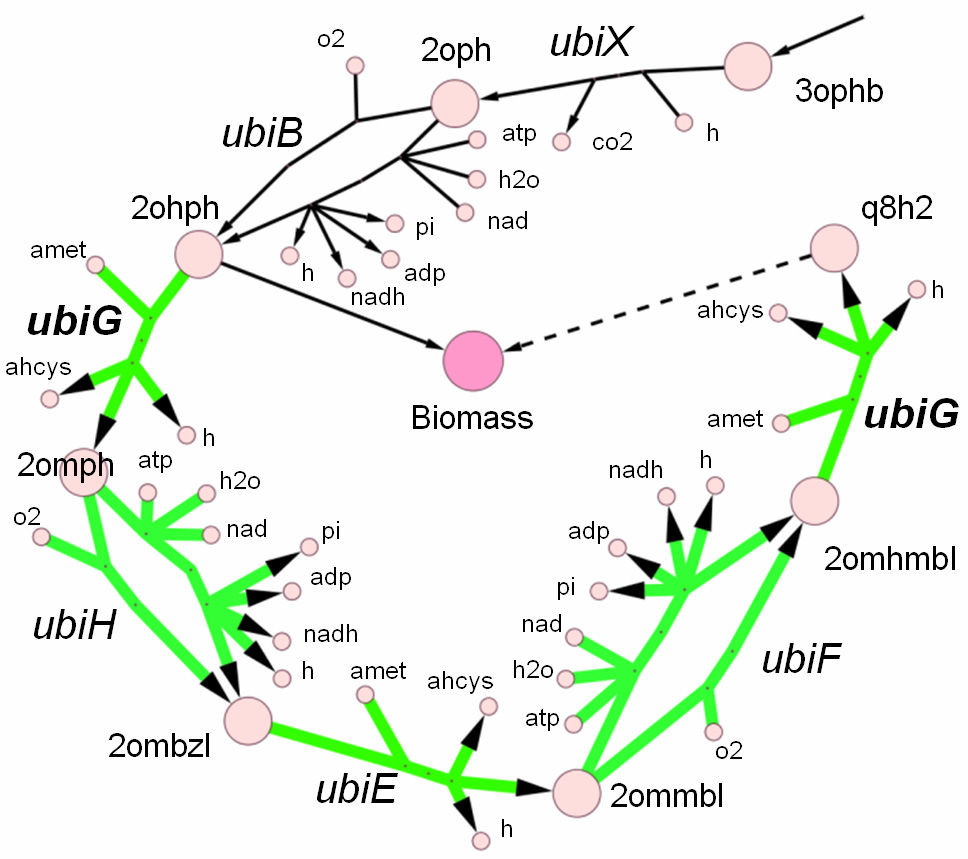
**Context-dependent activity of biosynthetic pathways for the co-factor ubiquinone-8 (q8h2)**. Edges represent reactions, circular nodes represent metabolites. Black (thin) edges represent reactions predicted to be active both by FBA and by MD-FBA and green (thick) edges represent reactions predicted to be inactive by FBA and active by MD-FBA. MD-FBA correctly predicts the pathway to be activated under succinate minimal medium (where q8h2 is used in aerobic respiration) and to be inactivated under other media. FBA falsely predicts the inactivity of the pathway (downstream to 2ohph), as it does not account for the dilution demand for the production of q8h2, which is not included in its biomass reaction (as it is used only under some environments). 2ohph, 2-octaprenyl-6-hydroxyphenol; 2ombzl, 2-octaprenyl-6-methoxy-1,4-benzoquinol; 2omhmbl, 2-octaprenyl-3-methyl-5-hydroxy-6-methoxy-1,4-benzoquinol; 2ommbl, 2-octaprenyl-3-methyl-6-methoxy-1,4-benzoquinol; 2omph, 2-octaprenyl-6-methoxyphenol; 2oph, 2-octaprenylphenol; 3ophb, 3-octaprenyl-4-hydroxybenzoate; ahcys, s-adenosyl-l-homocysteine; amet, s-adenosyl-l-methionine; atp, adenosine-3-phosphate; co2, carbon dioxide; h, hydrogen; h2o, water; nad, nicotinamide-adenine-dinucleotide; nadh, nicotinamide-adenine-dinucleotide-reduced; o2, oxygen; pi, phosphate; q8h2, ubiquinone-8-reduced.

Extending the gene essentiality analysis under these media for other genes, not included in the ASAP dataset, revealed several additional scenarios in which MD-FBA and FBA predictions significantly differ. We found that, generally, MD-FBA predicts the activation of reactions involved in co-factor biosynthesis that are not activated by FBA (the distribution of reactions whose predicted activity pattern differ between MD-FBA and FBA across various metabolic subsystems is shown in Additional file 3). For example, under succinate minimal medium, MD-FBA predicts that genes in the ubiquinone-8 biosynthetic pathway are essential for growth, whereas FBA predicts these genes to be nonessential (Figure 3). Specifically, both methods predict that the first part of this pathway, leading to the production of the biomass metabolite 2-octaprenyl-6-hydroxyphenol (black solid edges), is essential under succinate minimal medium, while only MD-FBA predicts that the remaining part of the pathway, leading to ubiquinone-8, is activated. Ubiquinone-8 is an important redox co-factor in *E. coli*'s aerobic respiration, switching between a reduced (q8h2) state and an oxidized (q8) state. While both FBA and MD-FBA predict the cycling of ubiquinone-8 between the reduced and oxidized states under succinate minimal medium (as part of aerobic respiration), only MD-FBA predicts the corresponding requirement for de novo synthesis of this metabolite to accommodate for its growth-associated dilution. Notably, this scenario is similar to that described in the toy model in Figure 1a, where q8 and q8h2 correspond to co-factor metabolites *C *and C*. As a testimony to the correctness of these predictions, we found that a gene coding for an enzyme catalyzing two reactions in the ubiquinone-8 biosynthetic pathway, *ubiG*, was experimentally validated to be essential for *E. coli *growing under succinate minimal medium [30,31]. Adding ubiquinone-8 to the biomass reaction would indeed solve the false essentiality prediction of *ubiG *under succinate minimal medium, but would lead to a false essentiality prediction under glucose medium, where *ubiG *was shown to be nonessential for growth [32] - further emphasizing the advantage of accounting for metabolite dilution in FBA.

**Figure 4:**
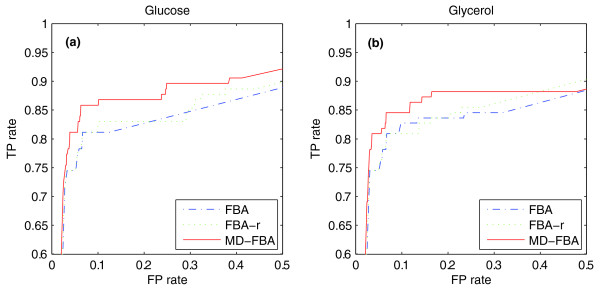
**ROC curves of gene essentiality predictions**. ROC curves of gene essentiality predictions under **(a) **glucose and **(b) **glycerol minimal media. Predictions were made by FBA, FBA-reduced biomass (FBA-r; utilizing the reduced biomass definition) and MD-FBA, where MD-FBA is shown to outperform both FBA and FBA-r under both growth media. FP, false positive; TP, true positive.

As an additional validation, we applied MD-FBA to predict gene essentiality for 1,117 genes under glucose and glycerol minimal media, based on measurements from [32] and [33], respectively. Each gene in the dataset was experimentally determined to be either essential or non-essential and the accuracy of the essentiality predictions obtained by FBA and MD-FBA was assessed via an area under curve (AUC) score of the receiver operating characteristic (ROC) curve [34]. This curve represents the true positive and false positive rates as a function of the threshold on the predicted growth rate used to determine gene essentiality (experimental and predicted datasets are available in Additional file 4). Initially, we applied MD-FBA, utilizing the same definition of a biomass as in FBA (as performed above), obtaining very similar AUC scores of 0.888/0.873 and 0.873/0.875 for MD-FBA and FBA, respectively, under glucose/glycerol. However, following further inspection, we found that 15 of the 63 metabolic precursors that make up the biomass are actually designated as co-factors by Feist *et al. *[6]; hence, MD-FBA is likely to be able to predict the growth-associated demand for their synthesis specifically under the conditions in which they are required, without accounting for them explicitly in the biomass definition. For example, in Figure 1, the dilution of co-factor *C *is correctly predicted by MD-FBA in a context-dependent manner only when metabolite *X *is present in the medium, as *C *is not fixed to be included in the biomass. Falsely including metabolite *C *in the biomass, although it is required in only some media, would lead to a false prediction of lethality when metabolite *X *is absent from the growth medium. Given that such an inclusion of co-factors in the biomass may lead to false gene essentiality predictions, their removal from the biomass is likely to improve prediction performance. In order to remove these co-factors from the biomass, we performed the following pre-processing step: in each growth condition examined, each co-factor was in turn removed from the biomass and MD-FBA was then applied to test whether a dilution is predicted for the co-factor under a subset of the gene knockout strains. The analysis revealed three co-factors (10-formyltetrahydrofolate, 2-octaprenyl-6-hydroxyphenol, flavin adenine dinucleotide oxidized (FAD)) whose dilution is dynamically predicted by MD-FBA and they were subsequently removed from MD-FBA's biomass (dilution analysis results are available in Additional file 5). Repeating the gene essentiality analysis with the reduced biomass considerably improved the prediction performance of MD-FBA (Figure 4). Specifically, the AUC scores achieved by MD-FBA and FBA under glucose medium are 0.910 and 0.873, respectively, and under glycerol medium are 0.893 and 0.875, respectively. As further support for the assertion that the improved prediction performance is not a mere consequence of removing unnecessary biomass precursors, we re-applied FBA using the same reduced biomass (labeled FBA-r in Figure 4), which showed no improvement over FBA's original performance. These results clearly demonstrate the added-value of considering the context-dependent nature of co-factor requirements, which can change depending on both genetic and environmental factors.

## Discussion

This study presents MD-FBA, a variant of FBA for predicting metabolic flux distributions by accounting for growth-associated dilution of all metabolites in a context-dependent manner. The method predicts feasible flux distributions maximizing the production rate of a predefined biomass while accounting for the dilution of all intermediate metabolites, and most importantly, for all metabolic co-factors involved in the process. MD-FBA was shown to successfully predict *E. coli*'s gene essentiality under a variety of growth media and knockout strains, displaying a significant improvement upon the prediction performance of the commonly used FBA method.

MD-FBA has two notable limitations, which may contribute to the relatively low improvement in growth rate prediction accuracy (compared to the marked advantage in predicting gene knockout lethality). First, MD-FBA employs a uniform lower bound on the dilution rate of intermediate metabolites which, along with the absence of reactions outside the scope of the network model that degrade intermediate metabolites, implicitly reflects the assumption of a uniform concentration of all intermediate metabolites. A natural extension of MD-FBA would be to consider different lower and upper bounds on concentrations of different metabolites, based on concentration statistics gathered via metabolomic measurements across a variety of conditions (for example, [24]). Notably though, changing the lower bound employed here to a range of possible values and incorporating an upper bound on dilution rates across all metabolites did not improve the prediction performance (data not shown). Second, MD-FBA, similarly to FBA, is based on the assumption that microbial species aim to maximize their growth rate and hence search for feasible flux distributions that maximize biomass synthesis rate. However, previous studies have questioned this hypothesis, suggesting alternative possible optimization criteria. Future studies should investigate the potential usage of such optimization criteria with MD-FBA [35]. More generally, CBM methods that do not rely on optimization may also benefit from variants that account for metabolite dilution during growth.

A marked disadvantage of MD-FBA is its dependence on MILP, which is computationally more demanding than LP, utilized by FBA. To improve the run-time of MD-FBA, the amount of integer variables in the MD-FBA formulation may be reduced by employing a previous method to identify the metabolic 'scope' of the medium nutrients. Specifically, Handorf *et al. *[36] investigated the capacity to produce metabolites from available medium nutrients by applying FBA and a network expansion algorithm, resulting in a production scope for each set of medium metabolites. A potential improvement in run-time may be achieved by calculating the scope of the input growth medium and assigning integer variables only for metabolites in that derived scope, as all the other metabolites will never be able to satisfy their dilution demand. Speeding up the run-time may be of importance when applying MD-FBA to larger networks, such as the recently published human model [16], or when probing the network under multiple knockout configurations [37,38].

An interesting comparison can be made between MD-FBA and a method developed by Price *et al. *[39] for eliminating futile cycles via the identification of type III extreme pathways (that is, a unique set of convex basis vectors of the flux distribution solution space that do not include exchange reactions). While the extreme pathways method enables the elimination of thermodynamically impossible loops, MD-FBA removes infeasible solutions due to dilution demands. Notably, the latter method also implicitly eliminates type III extreme pathways since these pathways do not satisfy dilution demands of the participating metabolites. Additionally, MD-FBA eliminates solutions that do not involve type III extreme pathways as demonstrated in Figure 1b: when metabolite *X *is absent from the growth medium, the cycle involving reactions *v*_4 _and *v*_8 _cannot be activated based on MD-FBA, since the dilution of co-factor *C *cannot be satisfied, although this cycle is not part of a type III extreme pathway.

Another appealing application of MD-FBA could be the identification of missing reactions in the model by comparing predicted phenotypes with measured ones, in line with previous works using FBA for this purpose [40]. For example, suppose that in Figure 1a the biosynthetic pathway for metabolite *C*, through reactions *v*_6 _and *v*_7_, was not included in the model. In this case, MD-FBA would predict metabolic flow through reactions *v*_2 _and *v*_3_, such that the enzymes catalyzing these reactions are essential, contrary to experimental essentiality data. Utilizing a method similar to that used by Reed *et al. *[40], using MD-FBA can infer the missing reactions, *v*_6 _and *v*_7_. Employing FBA for this purpose would not work since FBA predicts *v*_2 _and *v*_3 _to be non-essential, as the activity of reactions *v*_4 _and *v*_8 _do not depend on the presence of reactions *v*_6 _and *v*_7_.

While this work applied MD-FBA to predict metabolic phenotypes in *E. coli*, for which a comprehensive and accurate metabolic network model exists, the method can also be applied to any one of a growing number of reconstructed network models [20]. Importantly, the application of MD-FBA to other network models is straightforward and requires no model-specific data curation. To facilitate simple usage of MD-FBA, we provide an implementation of the method in the supplemental website [41]. A particularly interesting potential application of MD-FBA would be for modeling malignant proliferating cells in human cancer, potentially revealing the activity of biosynthetic pathways for various co-factors required to balance their growth-associated dilution. The latter may utilize the recently published model of human cellular metabolism by [16] or [42]. Overall, we expect that future use of MD-FBA will promote improved metabolic phenotypic predictions across a variety of organisms, growth conditions and genetic alterations.

## Materials and methods

### Metabolite dilution flux balance analysis

To formulate a mass-balance constraint while accounting for metabolite growth dilution, we assume that each metabolite *j *that is produced by a certain reaction at a rate greater than zero (referred to as an 'active metabolite') has a non-zero concentration and should hence be diluted with a rate greater than zero (denoted by *d*_*j*_). To compute a feasible flux distribution, , and a corresponding vector of dilution rates, , we employ the following optimization problem:

subject to(3)(4)(5)

where a mass-balance constraint, accounting for the dilution of all active metabolites, is formulated in Equation 3. Equation 4 assigns a positive dilution rate above a pre-defined threshold (denoted by *ε*) for active metabolites, produced in some non-zero rate in the flux distribution . In our application of the method for *E. coli *we set *ε *= 10^-4 ^μmol/mg, which represents a common concentration of intermediate metabolites [6]. Notably, the model's predictions were robust to different choices of *ε *values (data not shown). Enzyme directionality and capacity constraints are formulated in Equation 5 by imposing  and  as lower and upper bounds on flux values.

The above optimization problem is solved by formulating it as a MILP problem, replacing the Equation 4 constraint with the linear equations specified below: for each metabolite *j *in the model, we define an integer variable *y*_*j *_that denotes whether the metabolite is active (that is, being produced by some non-zero reaction in the model), via the following linear constraints:(6)(7)

where *R*_*j *_denotes the set of reactions in which metabolite *j *participates. Equation 6 is a linear formulation of the statement *'if ν*_*i *_≥ *ε *then *y*_*j *_= 1' and Equation 7 is the symmetric for negative fluxes (that is, *ν*_*i *_≤ -*ε*). Given the  variables, Equation 4 can be formulated via the following constraints:(8)

which can be represented in linear form (since *εμ *< 1) as:

A simplified formulation assuming a constant growth rate of *μ *= 1 in Equation 8 (for calculating the dilution rate of intermediate metabolites) gave qualitatively similar results to the above linear formulation (data not shown). The commercial solver CPLEX running on 64-bit Linux machines was used for solving LP and MILP problems within a few dozens of seconds per problem.

## Abbreviations

AUC: area under curve; CBM: constraint-based modeling; FBA: flux balance analysis; LP: linear programming; MD-FBA: metabolite dilution flux balance analysis; MILP: mixed integer linear programming; OD: optical density; q8: ubiquinone-8-oxidized; q8h2: ubiquinone-8-reduced; ROC: receiver operating characteristic.

## Authors' contributions

TB developed the method, implemented the method, performed analyses and wrote the manuscript. OF performed analyses. ER conceived the study. TS conceived the study, developed the method and wrote the manuscript. All authors discussed the results, and read, commented and approved the final manuscript.

## Supplementary Material

Additional file 1 **ASAP growth conditions**. Excel file including the growth conditions used to model the ASAP experiments used by both FBA and MD-FBA. Click here for file

Additional file 2 **ASAP experimental optical density values**. Excel file including the experimentally measured OD values taken from the ASAP database. Click here for file

Additional file 3 **Reaction activity difference across subsystems**. Figure showing the mean reaction activity difference between FBA and MD-FBA across the different metabolic subsystems available in the model. Click here for file

Additional file 4 **Gene essentiality analysis results**. Excel file containing the gene essentiality predictions by FBA and MD-FBA as well as the experimental gene essentiality data. Click here for file

Additional file 5 **Cofactor dilution-related demand activity predictions**. Excel file containing MD-FBA's predictions for the dilution related growth-demand synthesis of the three cofactors: 10-formyltetrahydrofolate (10fthf), 2-octaprenyl-6-hydroxyphenol (2ohph) and FAD across different gene knockouts. Click here for file

## References

[B1] Fell DA Understanding the Control of Metabolism 1996 London: Portland Press

[B2] Domach MM Leung SK Cahn RE Cocks GG Shuler ML Computer model for glucose-limited growth of a single cell of *Escherichia coli *B/r-A. Biotechnol Bioeng 2000 67 827 840 10699861 10.1002/(sici)1097-0290(20000320)67:6<827::aid-bit18>3.0.co;2-n

[B3] Price ND Reed JL Palsson BO Genome-scale models of microbial cells: evaluating the consequences of constraints. Nat Rev Microbiol 2004 2 886 897 15494745 10.1038/nrmicro1023

[B4] Stelling J Klamt S Bettenbrock K Schuster S Gilles E Metabolic network structure determines key aspects of functionality and regulation. Nature 2002 420 190 193 12432396 10.1038/nature01166

[B5] Oberhardt MA Palsson BO Papin JA Applications of genome-scale metabolic reconstructions. Mol Syst Biol 2009 5 320 19888215 10.1038/msb.2009.77 PMC2795471

[B6] Feist AM Henry CS Reed JL Krummenacker M Joyce AR Karp PD Broadbelt LJ Hatzimanikatis V Palsson BO A genome-scale metabolic reconstruction for *Escherichia coli *K-12 MG1655 that accounts for 1260 ORFs and thermodynamic information. Mol Syst Biol 2007 3 121 17593909 10.1038/msb4100155 PMC1911197

[B7] Mo M Palsson B Herrgard M Connecting extracellular metabolomic measurements to intracellular flux states in yeast. BMC Syst Biol 2009 3 37 19321003 10.1186/1752-0509-3-37 PMC2679711

[B8] Durot M Le Fevre F de Berardinis V Kreimeyer A Vallenet D Combe C Smidtas S Salanoubat M Weissenbach J Schachter V Iterative reconstruction of a global metabolic model of *Acinetobacter baylyi *ADP1 using high-throughput growth phenotype and gene essentiality data. BMC Syst Biol 2008 2 85 18840283 10.1186/1752-0509-2-85 PMC2606687

[B9] Senger RS Papoutsakis ET Genome-scale model for *Clostridium acetobutylicum*: Part I. Metabolic network resolution and analysis. Biotechnol Bioeng 2008 101 1036 1052 18767192 10.1002/bit.22010 PMC2760220

[B10] Izallalen M Mahadevan R Burgard A Postier B Didonato R Sun J Schilling CH Lovley DR *Geobacter sulfurreducens *strain engineered for increased rates of respiration. Metab Eng 2008 10 267 275 18644460 10.1016/j.ymben.2008.06.005

[B11] Mahadevan R Bond DR Butler JE Esteve-Nunez A Coppi MV Palsson BO Schilling CH Lovley DR Characterization of metabolism in the Fe(III)-reducing organism *Geobacter sulfurreducens *by constraint-based modeling. Appl Environ Microbiol 2006 72 1558 1568 16461711 10.1128/AEM.72.2.1558-1568.2006 PMC1392927

[B12] Kjeld Raunkaer K Jens N *In silico *genome-scale reconstruction and validation of the *Corynebacterium glutamicum *metabolic network. Biotechnol Bioeng 2009 102 583 597 18985611 10.1002/bit.22067

[B13] Jamshidi N Palsson B Investigating the metabolic capabilities of *Mycobacterium tuberculosis *H37Rv using the *in silico *strain iNJ661 and proposing alternative drug targets. BMC Syst Biol 2007 1 26 17555602 10.1186/1752-0509-1-26 PMC1925256

[B14] Schilling C Covert M Famili I Church G Edwards J Palsson B Genome-scale metabolic model of *Helicobacter pylori *26695. J Bacteriol 2002 184 4582 4593 12142428 10.1128/JB.184.16.4582-4593.2002 PMC135230

[B15] Becker S Palsson B Genome-scale reconstruction of the metabolic network in *Staphylococcus aureus *N315: an initial draft to the two-dimensional annotation. BMC Microbiol 2005 5 8 15752426 10.1186/1471-2180-5-8 PMC1079855

[B16] Duarte NC Becker SA Jamshidi N Thiele I Mo ML Vo TD Srivas R Palsson BO Global reconstruction of the human metabolic network based on genomic and bibliomic data. Proc Natl Acad Sci USA 2007 104 1777 1782 17267599 10.1073/pnas.0610772104 PMC1794290

[B17] Varma A Palsson B Metabolic capabilities of *Escherichia coli*. II. Optimal growth patterns. J Theor Biol 1993 165 503 522 10.1006/jtbi.1993.1202 21322280

[B18] Varma A Palsson B Stoichiometric flux balance models quantitatively predict growth and metabolic by-product secretion in wild-type *Escherichia coli *W3110. Appl Environ Microbiol 1994 60 3724 3731 7986045 10.1128/aem.60.10.3724-3731.1994 PMC201879

[B19] Kauffman K Prakash P Edwards J Advances in flux balance analysis. Curr Opin Biotechnol 2003 14 491 496 14580578 10.1016/j.copbio.2003.08.001

[B20] Feist AM Herrgard MJ Thiele I Reed JL Palsson BO Reconstruction of biochemical networks in microorganisms. Nat Rev Microbiol 2009 7 129 143 19116616 10.1038/nrmicro1949 PMC3119670

[B21] Visser D Schmid JW Mauch K Reuss M Heijnen JJ Optimal re-design of primary metabolism in *Escherichia coli *using linlog kinetics. Metab Eng 2004 6 378 390 15491866 10.1016/j.ymben.2004.07.001

[B22] Pramanik J Keasling JD Stoichiometric model of *Escherichia coli *metabolism: incorporation of growth-rate dependent biomass composition and mechanistic energy requirements. Biotechnol Bioeng 1997 56 398 421 18642243 10.1002/(SICI)1097-0290(19971120)56:4<398::AID-BIT6>3.0.CO;2-J

[B23] Pramanik J Keasling JD Effect of *Escherichia coli *biomass composition on central metabolic fluxes predicted by a stoichiometric model. Biotechnol Bioeng 1998 60 230 238 10099424 10.1002/(sici)1097-0290(19981020)60:2<230::aid-bit10>3.0.co;2-q

[B24] Bennett BD Kimball EH Gao M Osterhout R Van Dien SJ Rabinowitz JD Absolute metabolite concentrations and implied enzyme active site occupancy in *Escherichia coli*. Nat Chem Biol 2009 5 593 599 19561621 10.1038/nchembio.186 PMC2754216

[B25] Feist AM Palsson BO The growing scope of applications of genome-scale metabolic reconstructions using *Escherichia coli*. Nat Biotechnol 2008 26 659 667 18536691 10.1038/nbt1401 PMC3108568

[B26] Kruse K Ebenhöh O Comparing flux balance analysis to network expansion: producibility, sustainability and the scope of compounds. Genome Informatics 2008 20 91 101 19425125

[B27] Martelli C De Martino A Marinaric E Marsili M Castilloe I Identifying essential genes in *Escherichia coli *from a metabolic optimization principle. Proc Natl Acad Sci USA 2009 106 2607 2611 19196991 10.1073/pnas.0813229106 PMC2636734

[B28] Covert MW Knight EM Reed JL Herrgard MJ Palsson BO Integrating high-throughput and computational data elucidates bacterial networks. Nature 2004 429 92 96 15129285 10.1038/nature02456

[B29] Glasner Jea ASAP, a systematic annotation package for community analysis of genomes. Nucleic Acids Res 2003 31 147 151 12519969 10.1093/nar/gkg125 PMC165572

[B30] Wu G Williams HD Zamanian M Gibson F Poole RK Isolation and characterization of *Escherichia coli *mutants affected in aerobic respiration: the cloning and nucleotide sequence of ubiG: Identification of an S-adenosylmethionine-binding motif in protein, RNA, and small-molecule methyltransferases. J Gen Microbiol 1992 138 2101 2112 1479344 10.1099/00221287-138-10-2101

[B31] Hsu AY Poon WW Shepherd JA Myles DC Clarke CF Complementation of coq3 mutant yeast by mitochondrial targeting of the *Escherichia coli *UbiG polypeptide: evidence that UbiG catalyzes both O-methylation steps in ubiquinone biosynthesis. Biochemistry 1996 35 9797 9806 8703953 10.1021/bi9602932

[B32] Baba T Ara T Hasegawa M Takai Y Okumura Y Baba M Datsenko KA Tomita M Wanner BL Mori H Construction of *Escherichia coli *K-12 in-frame, single-gene knockout mutants: the Keio collection. Mol Syst Biol 2006 2 2006.0008 16738554 10.1038/msb4100050 PMC1681482

[B33] Joyce A Reed J White A Edwards R Osterman A Baba T Mori H Lesely S Palsson B Agarwalla S Experimental and computational assessment of conditionally essential genes in *Escherichia coli*. J Bacteriol 2006 188 8259 8271 17012394 10.1128/JB.00740-06 PMC1698209

[B34] Bradley AP The use of the area under the ROC curve in the evaluation of machine learning algorithms. Pattern Recognition 1997 30 1145 1159

[B35] Schuetz R Kuepfer L Sauer U Systematic evaluation of objective functions for predicting intracellular fluxes in *Escherichia coli*. Mol Syst Biol 2007 3 119 17625511 10.1038/msb4100162 PMC1949037

[B36] Handorf T Ebenhoh O Heinrich R Expanding metabolic networks: scopes of compounds, robustness, and evolution. J Mol Evol 2005 61 498 512 16155745 10.1007/s00239-005-0027-1

[B37] Burgard A Maranas C Optimization-based framework for inferring and testing hypothesized metabolic objective functions. Biotechnol Bioeng 2003 82 670 677 12673766 10.1002/bit.10617

[B38] Deutscher D Meilijson I Kupiec M Ruppin E Multiple knockout analysis of genetic robustness in the yeast metabolic network. Nat Genet 2006 38 993 998 16941010 10.1038/ng1856

[B39] Price ND Famili I Beard DA Palsson B Extreme pathways and Kirchhoff's second law. Biophys J 2002 83 2879 2882 12425318 10.1016/S0006-3495(02)75297-1 PMC1302372

[B40] Reed JL Patel TR Chen KH Joyce AR Applebee MK Herring CD Bui OT Knight EM Fong SS Palsson BO Systems approach to refining genome annotation. Proc Natl Acad Sci USA 2006 103 17480 17484 17088549 10.1073/pnas.0603364103 PMC1859954

[B41] MD-FBA supplemental material http://www.cs.technion.ac.il/~tomersh/tools

[B42] Ma H Sorokin A Mazein A Selkov A Selkov E Demin O Goryanin I The Edinburgh human metabolic network reconstruction and its functional analysis. Mol Syst Biol 2007 3 135 17882155 10.1038/msb4100177 PMC2013923

